# Supplementary material for: Gap junctions deliver malonyl-CoA from soma to germline to support embryogenesis in Caenorhabditis elegans
Source: eLife. 2020 Jul 31;9:e58619. doi: 10.7554/eLife.58619 (PMC7445009; doi:10.7554/eLife.58619)
Supplement: Figure 1—source data 1. [file elife-58619-fig1-data1.docx]

**Figure 1–source data 1**

***inx-8(rf)* exhibits reduced brood size and interacts with FA synthesis pathway genes**

**________________________________________________________________**

Genotype Brood size Germ cells per gonad arm

**________________________________________________________________**

20^o^C

N2 (wild type) 291 ± 29 (n=33) ≥1000^1^

*inx-8(rf)*^2^ 108 ± 40 (n=90) 322 ± 18 (n=3; day 2 adult)

22^0^C

*inx-8(+)*^3^ 266 ± 40 (n=32)

*+/inx-8(rf)^4^*  255 ± 36 (n=32)

*fasn-1(g43ts); inx-8(+)* 115 ± 41 (n=11)

*fasn-1(g43ts); +/ inx-8(rf)* 114 ± 47 (n=11)

*fasn-1(g43ts); inx-8(rf)* 4.5 ± 2.3 (n=12)

*emb-8(hc69ts); inx-8(+)* 95 ± 50 (n=15)

*emb-8(hc69ts); +/inx-8(rf)* 35 ± 37 (n=15)

*emb-8(hc69ts); inx-8(rf)* 11 ± 9 (n=15)

__________________________________________________________

^1^ McCarter J, Bartlett B, Dang T, Schedl T. 1997. Soma-germ cell interactions in *Caenorhabditis elegans*: multiple events of hermaphrodite germline development require the somatic sheath and spermathecal lineages. *Developmental Biology* **181:**121–143. DOI: https://doi.org/10.1006/dbio.1996.8429, PMID: 9013925.

^2^Full genotype *inx-8(tn1513 tn1555) inx-9(ok1502null).* Approximately 4% of animals are infertile in one or both gonad arms (n=168).

^3^*inx-8(+)* provided on the balancer *mIs11* IV.

^4^Full genotype *mIs11/inx-8(tn1513 tn1555) inx-9(ok1502null).*

**Figure 1–source data 2.** Brood counts for Figure 1­–source data 1.
