# Supplementary material for: Gap junctions deliver malonyl-CoA from soma to germline to support embryogenesis in Caenorhabditis elegans
Source: eLife. 2020 Jul 31;9:e58619. doi: 10.7554/eLife.58619 (PMC7445009; doi:10.7554/eLife.58619)
Supplement: Figure 1—source data 3. [file elife-58619-fig1-data3.docx]

**Figure 1–source data 3**

**Reduced soma-germline gap junction coupling delays gametogenesis**

**(a) *gfp::lin-41* expression at L4 vulval sub-stages**

Genotype L4.4 L4.5 L4.6 L4.7 L4.8 L4.9 L4.9< < molt

*gfp::lin-41; inx-8(+)* 1/11 3/3 5/6 5/6 4/4

*gfp::lin-41; inx-8(rf)^1^*  0/2 0/3 0/6 0/2 16/37

*gfp::lin-41; inx-8/9(0);*  0/11 0/3 0/2 0/1 0/3 6/14

*Ex[inx-8(DTC+,Sh-)]*

**(b) sperm appearance at L4 sub-stages**

Genotype L4.4 L4.5 L4.6 L4.7 L4.8 L4.9 L4.9< < molt

N2 0/9 0/2 0/5 4/11 1/2 1/2 6/6

*inx-8(rf)^1^*  0/3 0/1 0/2 0/3 0/1 1/10

^1^Full genotype *inx-8(tn1513 tn1555) inx-9(ok1502null).*
