# Supplementary material for: Gap junctions deliver malonyl-CoA from soma to germline to support embryogenesis in Caenorhabditis elegans
Source: eLife. 2020 Jul 31;9:e58619. doi: 10.7554/eLife.58619 (PMC7445009; doi:10.7554/eLife.58619)
Supplement: Supplementary file 1. [file elife-58619-supp1.docx]

**Strain list**

Bristol N2

AG400 *fasn-1(av138[fasn-1::gfp]) I*

DG4153 *pod-2(tn1691null) II; tnEx212 [pod-2(+)—high (20 ng/μl); sur-5::gfp]*

DG4324 *pod-2(tn1765[gfp::pod-2]) II*

DG4329 *fasn-1(tn1762null) I/ hT2(qIs48) I:III*

DG4453 *emb-8(hc69ts) III; mIs11/ inx-8(tn1513 tn1555rf) inx-9(ok1502null) IV; tnEx212 [pod-2(+)—high (20 ng/μl); sur-5::gfp]*

DG4487 *fasn-1(tn1782[gfp::fasn-1]) I*

DG4527 *fasn-1(g43ts) I; mIs11/ inx-8(tn1513 tn1555rf) inx-9(ok1502null) IV; tnEx212 [pod-2(+)—high (20 ng/μl); sur-5::gfp]*

DG4531 *mIs11/ inx-8(tn1474null) inx-9(ok1502null) IV; tnEx205 [lag-2p::inx-8::gfp; str- 1::gfp]*

DG4702 *fasn-1(tn1762null) I/ hT2(qIs48) I:III; tnEx218 [fasn-1(+); sur-5::gfp(+)]*

DG4921 *inx-8(tn1513 tn1555rf) inx-9(ok1502null) IV*

DG4923 *pod-2(tn1691null) II; tnEx219 [pod-2(+)—low (4 ng/μl); sur-5::gfp]*

DG4924 *fasn-1(tn1762null) I; inx-8(tn1513 tn1555rf) inx-9(ok1502null) IV; tnEx218 [fasn-1(+); sur-5::gfp(+)]*

DG4925 *inx-8(tn1474null) inx-9(ok1502null) IV*; *tnEx221 [inx-8(tn1513 tn1555rf)::gfp; str-1::gfp]*

DG4929 *lin-41(tn1541[gfp::lin-41)] I; mIs11/ inx-8(tn1474null) inx-9(ok1502null) IV; tnEx205[lag-2p::inx-8::gfp; str-1::gfp]*

DG4930 *tmC18[dpy-5(tmIs1236)]/ fasn-1(tn1762null) I; tnEx218 [fasn-1(+); sur-5::gfp(+)]*

DG4931 *fasn-1(tn1762null) I/ hT2(qIs48) I:III; inx-8(tn1513 tn1555rf) inx-9(ok1502null) IV; tnEx218 [fasn-1(+); sur-5::gfp(+)]*

GG43 *fasn-1(g43ts) I*

HY520 *pod-2(ye60cs) II*

MJ69 *emb-8(hc69ts) III*
